# Phenolic Biotransformations in Wheatgrass Juice after Primary and Secondary Fermentation

**DOI:** 10.3390/foods12081624

**Published:** 2023-04-12

**Authors:** Baljinder Kaur, Balvir Kumar, Geetika Sirhindi, Nidhi Guleria, Jashandeep Kaur

**Affiliations:** 1 Systems Biology Laboratory, Department of Biotechnology and Food Technology, Punjabi University, Patiala 147002, Punjab, India; 2 Department of Biotechnology, University Institute of Biotechnology, Chandigarh University, Mohali 140413, Punjab, India; balvir.e10913@cumail.in; 3 Department of Botany, Punjabi University, Patiala 147002, Punjab, India; geetika@pbi.ac.in; 4 Department of Biotechnology and Food Technology, Punjabi University, Patiala 147002, Punjab, India; nidhiguleria1012@gmail.com (N.G.); jashangrewal312@gmail.com (J.K.)

**Keywords:** nutritional beverages, wheatgrass juice, lactic acid bacteria, metabolomic analysis, therapeutic phenols in fermented drinks

## Abstract

Fermented wheatgrass juice was prepared using a two-stage fermentation process by employing *Saccharomyces cerevisiae* and recombinant *Pediococcus acidilactici* BD16 (*alaD*^+^). During fermentation, a reddish-brown hue appeared in wheatgrass juice due to production of different types of red pigments. The fermented wheatgrass juice has considerably higher content of anthocyanins, total phenols and beta-carotenes as compared to unfermented wheatgrass juice. It has low ethanol content, which might be ascribed to the presence of certain phytolignans in wheatgrass juice. Several yeast-mediated phenolic transformations (such as bioconversion of coumaric acid, hydroxybenzoic acid, hydroxycinnamic acid and quinic acid into respective derivatives; glycosylation and prenylation of flavonoids; glycosylation of lignans; sulphonation of phenols; synthesis of carotenoids, diarylnonanoids, flavanones, stilbenes, steroids, quinolones, di- and tri-terpenoids and tannin) were identified in fermented wheatgrass juice using an untargeted liquid chromatography (LC)-mass spectrometry (MS)-matrix-assisted laser desorption/ionization (MALDI)-time-of-flight (TOF)/time-of-flight (TOF) technique. The recombinant *P*. *acidilactici* BD16 (*alaD*^+^) also supported flavonoid and lignin glycosylation; benzoic acid, hydroxycoumaric acid and quinic acid derivatization; and synthesis of anthraquinones, sterols and triterpenes with therapeutic benefits. The information presented in this manuscript may be utilized to elucidate the importance of *Saccharomyces cerevisiae* and *P*. *acidilactici* BD16 (*alaD*^+^) mediated phenolic biotransformations in developing functional food supplements such as fermented wheatgrass juice.

## 1. Introduction

The young grass of common wheat plant *Triticum aestivum* is generally referred to as wheatgrass. Wheatgrass belongs to the family Gramineae, class Liliopsida, order Cyperales, genus *Triticum* and species *Triticum aestivum* [1]. Wheatgrass juice is extracted from small wheat sprouts obtained after 6-10 days of germination. Wheatgrass juice is generally considered a powerhouse of amino acids (alanine, arginine, aspartic acid, glutamic acid and serine) and chlorophylls [2]. Wheatgrass juice contains handsome proportions of health promoting compounds such as dietary fibres; vitamins A, B, C and E; minerals such as calcium, phosphorus, magnesium; alkaline earth metals such as potassium, zinc, boron and molybdenum; and enzymes including amylase, cytochrome oxidase, lipase, protease, super-oxide dismutase and trans-hydrogenase [1,3]. Therefore, the consumption of wheatgrass juice is considered an energy booster [2].

Wheatgrass juice, popularly known as “green blood” is used in “green blood therapy”. Patients with chronic allergies, atherosclerosis, asthma, bronchitis, constipation, diabetes, eczema, hypertension, insomnia, joint pain and tuberculosis are advised to try wheatgrass therapy [2]. In a clinical trial, 15 female patients suffering from severe rheumatoid arthritis were administered with standard disease-modifying anti-rheumatic drug (DMARD) and steroid therapies, while receiving 2× single dosage of Avemar^®^ per day as additional therapy. Avemar^®^ is a medical food manufactured by Hungarians that contains standardized fermented wheatgerm extract with approved oncological indications. Administration of Avemar^®^, a fermented wheat germ extract, decreased their Ritchie index, as well as joint pain and swelling. Patients showed significant improvement from morning stiffness and their steroid dependency also reduced to half [4]. In another clinical study, wheatgrass tablets were given to 40 children (aged between 2 to >8 years) suffering from Thalassemia major on an empty stomach for a period of at least one year in divided doses of two to eight tablets per day (500 mg per tablet). This treatment was reported to provide health benefits by improving their Hb levels, increasing intervals required between two consecutive blood transfusions and decreasing the amount of blood required for transfusion [5]. A clinical investigation was also performed on 59 female patients suffering from atherogenic lipoproteins, blood sugar, inflammation and menopausal symptoms. The administration of freeze-dried wheatgrass powder (at a dose of 3.5 g daily for 10 weeks) was reported to decrease Apo B fraction, TC and TAG levels significantly in the intervention group (*n* = 29) as compared to control group (*n* = 30) [6].

The antioxidant components of wheatgrass juice display vivid biological activities such as prevention of oxidative damage to DNA and lipids, inhibition of carcinogen formation, stimulation of gap junction communication, inhibition of cancer cell proliferation, promotion of cellular differentiation and apoptosis and activation of innate and adaptive immune functions [7,8]. Drinking wheatgrass juice is recommended to cancer patients during chemotherapy because it can help to develop a healthier blood level and to reduce the requirement of blood-building drugs [2]. The intake of chlorophyll rich wheatgrass juice was found to be very effective in the treatment of skin infections and colon and skin ulcers [2,9,10]. Wheatgrass juice, due to its anti-microbial properties, is an excellent candidate for inclusion in mouthwashes for preventing pyorrhoea and sore throats [3].

Probiotic lactic acid bacteria (LAB) have a well evidenced history of utilization in various food, feed and pharmaceutical ingredients [11]. Moreover, interesting exogenous genetic attributes can be added by employing advanced genetic engineering approaches, such as metabolic engineering or enzyme engineering, to further widen their prospects for food utilization [12,13]. Keeping this in mind, a natural chilly pickle isolate, namely *Pediococcus acidilactici* BD16 (MTCC 10973), was chosen for the expression of synthetic *alaD* gene cassette encoding alanine dehydrogenase enzyme (AlaDH) using pLES003 shuttle vector [14,15]. AlaDH is capable of catalysing reductive amination of pyruvate into L-alanine, which is an important food additive with distinct biomedical activities, such as anti-bacterial, anti-cancer and anti-urolithiatic [16]. The developed strain was used to perform secondary fermentation of ginger, kiwi, plum and rose wines. Using GC-MS-based metabolic profiling, notable metabolic biotransformations leading to the enhanced production of 4-amino-1-pentanol, 2-aminononadecane, actinobolin, L-alanine, octanoic acid, 8-azononane, heptadecanenitrile, 3-butynol, adamentanemethylamine, benzeneethanamine, etc., which were found to be responsible for improving attributes of fermented beverages, were displayed [17]. This proof of concept was carried forward for developing fermented wheatgrass juice with an objective to demarcate the importance of *P. acidilactici* BD16 (*alaD*^+^) assisted metabolic transformations during fermentation of wheatgrass juice and enhancement of its qualitative attributes.

## 2. Materials and Methods

### 2.1. Procurement of Wheatgrass and Extraction of Wheatgrass Juice

Wheatgrass was procured from a local vendor, near Rajpura, Punjab, India. The fresh wheatgrass was rinsed with distilled water to remove any extraneous matter and then treated with 600 ppm potassium metabisulfite (KMS) for 15 min. Approximately, 1 litre of wheatgrass juice was extracted by grinding 2 kg wheatgrass using a mechanical mixer grinder (Philips kitchen appliance) and clarified by passing through muslin cloth. No water or chemical preservative was used for the extraction and preservation of wheatgrass juice. In a 2 L Erlenmeyer flask, 1000 mL wheatgrass juice and sugar solution were added, and total sugar content was adjusted to 23 °Brix using a handheld refractometer. The pH was adjusted to 4.5 by addition of fresh lemon juice. The total volume was increased to 2 L by addition of Bisleri mineral water. After proper mixing by manual stirring, the contents of the Erlenmeyer flask were distributed equally (666 mL per flask) into three fermentation vessels of 1 L capacity each to perform primary fermentation.

### 2.2. Primary Fermentation Using S. cerevisiae

Dry baker’s yeast (*S. cerevisiae*) was procured from a grocery store at Punjabi University Patiala, Punjab, India. Baker’s yeast (500 ppm) was added to the vessels after activating in lukewarm water (35 °C). Flasks were covered with water plugs and manual shaking was performed intermittently for 3 days. Afterwards, the flasks were left undisturbed for 25–30 days at ambient temperature (25 °C). The completion of primary alcoholic fermentation was indicated by settling of the wheatgrass partials and yeast precipitates. After the completion of primary fermentation (27 days), fermented wheatgrass juice was clarified by filtration through muslin cloth. The filtered juice was transferred to clean vessels and left undisturbed for 10 to 15 days to allow further clarification, then the clarified fermented juice was transferred to another clean vessel. This process, known as racking, was performed 2–3 times to obtain clear fermented juice.

### 2.3. Secondary Fermentation Using Pedicococcus acidilactici BD16 (alaD+)

*P. acidilactici* BD16 (*alaD^+^*) was procured from Systems Biology Lab, Department of Biotechnology and Food Technology, Punjabi University Patiala, Punjab, India. It was revived and sub-cultured thrice in 500 mL Erlenmeyer flasks containing 300 mL sterile MRS media (prepared by mixing 20 g/L dextrose, 10 g/L beef extract, 10 g/L peptone, 5 g/L sodium acetate, 5 g/L yeast extract, 2 g/L tri-ammonium citrate, 2 g/L di-potassium hydrogen phosphate, 0.1 g/L magnesium sulfate, 0.05 g/L manganous sulfate, 20 µg/mL erythromycin and 1 mL/L tween 80, pH 6.5 ± 0.2 under microaerophilic and stationary conditions) at 37 °C for 24 h [15]. The freshly grown culture of *P. acidilactici* BD16 (*alaD^+^*) was centrifuged at 5000 rpm for 10 min at 4 °C to collect bacterial pellet. The bacterial pellet was washed thrice using 20 mL sterile saline (100 mM NaCl) to obtain a whitish-creamy pellet. Optical density of the inoculum was adjusted to 2.0 using sterile saline solution (against blank saline at 600 nm). Inoculum (2% *v/v*) was added to the secondary fermentation vessels each containing 100 mL fermented wheatgrass juice obtained after primary fermentation. The fermented wheatgrass juice was kept undisturbed at ambient temperature (25 °C) for 7 days and then clarified by filtration using muslin cloth. After settling of the bacterial pellet, racking was performed 2 to 3 times to obtain clear fermented wheatgrass juice. Thereafter, the fermented wheatgrass juices were stored at 4 °C in a refrigerator until further analysis.

### 2.4. Biochemical Analyses of Wheatgrass Juices after Primary and Secondary Fermentation

#### 2.4.1. Determination of Total Soluble Solids (TSS) and Moisture Content

The proportion of total soluble solids (TSS) was read using a hand-held refractometer as °Brix at the point where the demarcation line between bright and dark sections crosses the vertical scale [18]. Moisture content was estimated using following formula [19].

Total Moisture Content = 100−TSS of the sample

#### 2.4.2. Determination of Total Acids by Titration Method

The total acid content in different samples was determined in terms of percentage by titration against 0.1N NaOH in the presence of phenolphthalein as indicator [17].

Percentage of acid = N×V×M.Eq.×100/volume of sample (mL)

*N* = Normality of NaOH

*V* = Volume of sodium hydroxide used to reach the titration end point (mL)

*M. Eq.* (milli-equivalents of acid) = Molecular weight of the acetic acid /100

#### 2.4.3. Determination of Total Proteins

The total proteins present in different samples was estimated using standard Lowry method. To 1 mL fermented juice sample, 5 mL alkaline copper sulphate solution was added and mixed thoroughly. It was allowed to stand undisturbed for 10–15 min at room temperature, then 0.5 mL of Folin–Ciocalteau reagent was added to the samples. After stirring, it was incubated at room temperature (25 °C) for 30 min to allow the development of a blue-coloured complex whose absorbance was measured at 750 nm using UV-VIS spectrophotometer. The amount of total protein was quantified using Bovine Serum Albumin (BSA) calibration curve drawn for different concentrations of bovine serum albumin ranging from 100 to 1000 µg/mL [20].

#### 2.4.4. Determination of Ethanol Content

The ethanol content in fermented wheatgrass juice was determined using standard distillation procedure [21]. Sample was prepared by mixing 1 mL of fermented juice with 30 mL distilled water. Distillation was performed at 80 °C in a round bottom distillation flask and the condensate was collected in a 250 mL conical flask containing 25 mL dichromic solution (0.1 M solution prepared by dissolving 34 g potassium dichromate in 100 mL distilled water containing 325 mL of H_2_SO_4_ and volume was adjusted to 1000 mL). After collection of the condensate, sample was incubated in a water bath at 60 °C for 15 min and absorbance was measured at 600 nm using UV-VIS spectrophotometer against water as a blank. The ethanol content in different samples was estimated in terms of percentage using standard curve of ethyl alcohol drawn for alcohol concentrations ranging from 2 to 10% *v/v*.

#### 2.4.5. Determination of Total Phenol Content

The clarified 1 mL fermented juice was mixed with 1.0 mL Folin–Ciocalteau reagent. To the above mixture, 4.0 mL sodium carbonate solution (20% *w/v*) was added after 5 minutes of incubation at ambient temperature and then distilled water was added to make total volume 10 mL. Sample was incubated at room temperature for about 2 h and absorbance was measured at 765 nm. Total phenols were estimated using standard curve of gallic acid (concentration range 10–100 µg/mL) and calculated in terms of gallic acid equivalents-GAE [22].

#### 2.4.6. Determination of Total Flavonoid Content

The sample was prepared by mixing 0.5 mL of clarified fermented juice with 1.5 mL methanol. To the above sample, 0.1 mL of 10% aluminium chloride, 0.1 mL of 1 M potassium acetate and 2.8 mL of distilled water were added and mixed thoroughly. Absorbance of the sample was measured at 415 nm after incubating the reaction mixture at room temperature for 30 min. The total flavonoid content was estimated in terms of quercetin equivalents from the calibration curve drawn in the concentration range from 100 to 1000 µg/mL [23].

#### 2.4.7. Estimation of Total Anthocyanin Content

To the clarified fermented juice (4 mL), an equal amount of methanol solution (containing 60% methanol in water containing 1% HCl) was added and the total volume was increased to 10 mL using distilled water. The blank solution was prepared by mixing 4.8 mL of methanol solution and 5.2 mL of distilled water. The total anthocyanin content was estimated in terms of cyanidin-3-glucoside by measuring absorbance at 530 nm [24]. Total anthocyanin content is:

(1) (mg Cy3G/L)=A×M.W.× D.F.× 1000/∈× L

where, *A* = absorbance at 530 nm; ∈ = molar extinction coefficient for cyanidin-3-glucoside (26,900); *L* = path length (1 cm); *D.F.* = dilution factor; *M.W.* = molecular weight for cyanidin-3-glucoside (484.8).

#### 2.4.8. Determination of Different Pigments

For the estimation of total carotenoid content, a given amount of clarified sample was mixed with an equal volume of 80% acetone (1:1). The optical densities of chlorophyll A, chlorophyll B and carotenes were measured at 663 nm, 643 nm and 470 nm, respectively [25]. A solution of acetone: hexane was prepared by mixing acetone and hexane in the ratio of 4:6. To estimate the contents of β-carotene and lycopene in different samples, the acetone and hexane solution and fermented juice were mixed in equal proportions and absorbance was measured at 453 nm, 505 nm, 645 nm and 663 nm [26].

Total carotenoids (mg/L) = $100A_{470}-1.80A_{663}-85.02A_{643}/198$

Lycopene content (mg/L) = $-0.0458A_{663}+0.372A_{505}-0.0806A_{453}$

β-carotene content (mg/L) = $0.216A_{663}-0.304A_{505}+0.452A_{453}$

#### 2.4.9. Determination of Colour Intensity

The absorbance of sample was directly measured at 420 nm (% Ye for yellow or brown pigment mainly flavonoids, tannins and some anthocyanins), 520 nm (% Rd for red pigment, mostly anthocyanins) and 620 nm (% Bl for blue pigment, mostly anthocyanins) using an optical path length of 2 nm [27].

Tint of wine (redness of wine) is defined by ratio = $A_{420}/A_{520}$

Brilliance of wine, dA (%) = $1-{(A}_{420}+{A_{620}/A}_{520})\times 100$

### 2.5. Study of Phenolic Biotransformations in Fermented Wheatgrass Juice by Untargeted LC-MS MALDI-TOF/TOF Technique

The clarified wheatgrass juice samples were acidified to pH 2.0 using 6N HCl; further, solvent extraction was performed using an equal volume of ethyl acetate (1:1 ratio) to extract phenolics [28]. Samples were stirred overnight at a speed of 50 RPM on a rotary shaker. Then, the ethyl acetate fraction was collected by centrifugation at 3000 rpm for 5 min for further analysis by LC-MS-MALDI-TOF/TOF. For the metabolomic analysis, the SYNPT-XS HDMS machine (Waters) on the separation module UPLC Acquity H class series system was used. The samples were tested on C18 waters column (Acquity BEH 2.1 × 100 mm, particle size 1.7 µm) using an injector volume of 5 microlitres at Sophisticated Analytical Instrumentation Facility (SAIF), Punjab University, Chandigarh, India. A gradient mobile phase consisting of 0.1% formic acid in water (solvent A) and 0.1% formic acid in acetonitrile containing 10% water (solvent B) was applied to the column for LC-MS-MALDI-TOF/TOF analysis [29]. It was introduced in the system at a flow rate of 0.15 mL/min using the following solvent gradient: for 0 min 90/10, 2 min 90/10, 5 min 80/20, 10 min 70/30, 12 min 50/50 and 14 min 10/90 for solvents A and B, respectively. The nitrogen and argon supply were maintained at pressures of 6–7 bars and 5–6 bars, respectively. The following mass spectrometer conditions were adjusted during analysis: desolvation gas: 950 L/h, cone gas: 50 L/h, desolvation temperature: 450 °C, source temperature: 120 °C, capillary voltage: 3.22 keV, cone voltage: 50 V and collision energy: 4 eV. The chromatograms were obtained, and each major peak was further analysed for the identification of different phenolic compounds by comparison with NIST Mass Spectral Library (available at http://www.chemdata.nist.gov, access date on July 2022) and previous scientific reports.

## 3. Results

Baker’s yeast *Saccharomyces cerevisiae* and a lactic acid bacterium *Pediococcus acidilactici* BD16 (*alaD*+) were used for preparing fermented wheatgrass juice. After filtration and racking, clarified samples were collected and subjected to different biochemical investigations and untargeted metabolomic analysis by LC-MS-MALDI-TOF/TOF.

### 3.1. Biochemical Analysis of Fermented Wheatgrass Juice

The TSS of wheatgrass juice decreased from 23 to 6.2 °Brix after subjecting to two-stage fermentation procedures. As TSS and total moisture are inversely correlated, total moisture increased from 77.1 to 93.7% after fermentation. At the time of start, TSS of wheatgrass juice was adjusted to 23 °Brix, which decreased gradually due to conversion of sugar into alcohol by the fermenting yeast. The acidity of wheatgrass juice (0.2 ± 0.017%) gradually increased to 1.08 ± 0.66% after primary fermentation then dropped to 0.65 ± 0.069% after secondary fermentation. This might be due to the fact that the acidity of fermented wheatgrass juice was modulated after secondary fermentation by the lactate dehydrogenase activity of hetero-fermentative *P. acidilactici*. The total protein content of unfermented wheatgrass juice was 4.6 ± 0.101 mg/mL, while it was estimated to be 4.07 ± 0.325 mg/mL after primary fermentation and 4.52 ± 0.305 mg/mL after secondary fermentation. These variations can be attributed to the activity of yeast and lactic acid bacteria. The fermented wheatgrass juice has low ethanol content (3.7 ± 0.005%) compared to fruit wines, which under standard fermentation conditions can accumulate up to 14–15% ethanol [30]. This might be attributed to the presence of certain phytolignans such as austrabailignan-7, dihydroguaiaretic acid, fragransin D1 and pinobanksin arabinose in wheatgrass juice which have resulted in the inhibition of alcohol dehydrogenase activity of yeast [31]. It has also been documented previously that the ethanol content in wine depends on numerous biotic and abiotic factors such as genotypic features of the fermentative strains, chemical composition or the original sugar content of fruit or substrate, presence of inhibitory substances, occurrence of competing pathways, fermentation temperature and time period for which fermentation is carried out [32,33].

In the present study, it was observed that total phenols first increased during primary fermentation then decreased during LAB assisted secondary fermentation phase. This decrease in the total phenolic content after secondary fermentation is attributed to the conversion of phenols into polyphenols as described earlier [34]. The phenolic content of wheatgrass juice was 0.41 ± 0.01 mg GAE/mL, while it was estimated to be 1.45 ± 0.04 and 1.36 ± 0.03 mg GAE/mL in fermented wheatgrass juice after primary and secondary fermentation, respectively. Total flavonoid content in wheatgrass juice (0.39 ± 0.0105 mg QE/mL) decreased after yeast fermentation (0.17 ± 0.011 mg QE/mL); however, no distinct change in the flavonoids was reported after secondary fermentation (0.169 ± 0.0152 mg QE/mL). The total anthocyanin content of wheatgrass juice was 0.97 ± 0.166 mg Cy3G/L, which increased successively from 3.50 ± 0.67 mg Cy3G/L to 4.53 ± 0.057 mg Cy3G/L after primary and secondary fermentation, respectively. A moderate increase in the total carotenoid content of fermented wheatgrass juice was also reported. The total carotenoid content of wheatgrass juice (0.25 ± 0.017 mg/mL) increased (0.31 ± 0.09 mg/mL) after primary and secondary fermentation, with a concomitant increase in contents of beta-carotene and lycopene after two phase fermentation process. Due to production of red pigments by fermenting yeast including anthocyanins such as malvidin glycosides, pelarogonidin, peonidin glycosides; carotenoids such as zeaxanthin; tannin namely deoxyschisandrin; and tyrosine derivative betanin, a reddish-brown hue appeared in the fermented wheatgrass juice. The pigment also contributes to colour intensity of fermented wheatgrass juice, which increased progressively during both the fermentation phases. Table 1 shows the comparison of different biochemical properties observed in the fermented wheatgrass juice after primary and secondary fermentation.

### 3.2. Phenolic Biotransformations in Fermented Wheatgrass Juice

**Figure 1:**
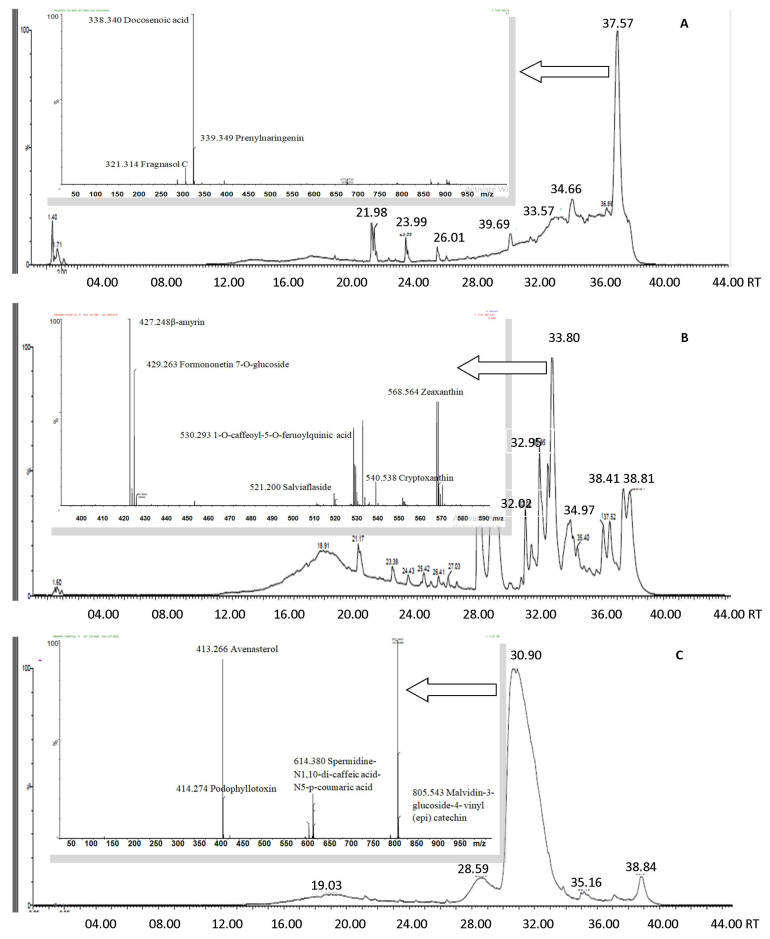
LC-MS-MALDI-TOF/TOF chromatograms of (**A**) wheatgrass juice, (**B**) wheatgrass juice after primary fermentation, and (**C**) wheatgrass juice after secondary fermentation. The x-axis denotes retention time (min) and y-axis denotes percentage intensity. Inset pictures show identified compounds in respective samples.

In the present study, untargeted LC-MS-MALDI-TOF/TOF has been utilized for the efficient and quick identification of phenolic derivatives present in fermented wheatgrass juice. TOF/TOF has permitted the mass analysis of metabolites by calculating their arrival time on the detector, with superior resolution. For the identification of individual compounds, their chromatographic retention times and parent ion masses were compared with the scientific literature and data contained in NIST Mass Spectral library (Figure 1). Table 2 lists different compounds detected in wheatgrass juice after primary and secondary fermentation using LC-MS-MALDI-TOF/TOF. Yeast has contributed to the development of bioactive phenolics such as 1-O-caffeoyl-β-D-glucose, 1-O-sinapoyl-β-D-glucose, 3-p-coumaroyl quinic acid, 6-C-hexosyl-chrysoeriol-O-rhamnoside-O-hexoside, 8-prenyl naringenin, β-amyrin, betanin, catechin, chrysoeriol-C-hexoside-C-pentoside, deoxyschisandrin, dihydrocaffeic acid 3-O-glucuronide, dihydroferulic acid sulphate, ellagic acid, esculin, gibberellin acid 8-hexose-gibberellin, kaempferol-rha-xyl-gal, laricitrin-3-O-rutinose, linoleic acid, malabaricone B, malvidin 3-O-rutinoside, naringenin glucuronide sulfate, naringenin sulfate, neoeriocitrin, p-coumaroyl-hexose-methylglutarate, pentacosenoic acid, pentahydroxy dimethoxy flavones, petunidin-3-O-glucoside, pinobanksin arabinose, pinocembrin-O-arabirosyl-glucoside, protocatechuic acid 4-O-glucoside, quercetin 7-O-malonylhexoside, quinic acid, salviaflaside derivatives, salvianolic acid, taxifolin-3-O-glucoside, trans-scirpusin A, trihydroxy-ent-kauranoic acid, vanillic acid 4-sulphate, violanone, vebonol and zeaxanthin after primary fermentation.

Secondary fermentation using *P. acidilactici* BD16 (*alaD*+) assisted in further augmentation of the therapeutic index of fermented wheatgrass juice. In the present study, important secondary metabolites (such as 2,4-dimethylphenol, apigenin-7-O-glucoside, avenasterol, fragransin D1, fraxetin-7-O-sulfate, fucosterol, luteolin-8-C-glucoside, malvidin-3-glucoside-4-vinyl (epi) catechin, myricetin 7-O-pentoside, myricetin 7-O-rhamnoside, neotigogenin acetate, peonidin 3-O-sambioside-5-O-glucoside, rhein, spermidine-N-1,10-di-caffeic acid-N5-p-coumaric acid and spermidine-N-5,10-di-p-coumaric acid-N-1-caffeic acid) were generated only after secondary fermentation, whereas wheatgrass metabolites (such as 2-methyl-4,6- dinitrophenol, apigenin-7-O-glucoside, avenasterol, fraxetin-7-O-sulfate, fucosterol and 2-hydroxy-4-methoxy-3,6-dimethylbenzoic acid) were also regenerated in the secondary fermentation process. Application of two-stage fermentation process resulted in development of secondary metabolites with already proven therapeutic activities (such as anti-cancerous, anti-diabetic, anti-inflammatory, anti-microbial, anti-oxidant, anti-viral, cardioprotective, gastroprotective, hepatoprotective, neuroprotective and osteoprotective), in addition to enhancement of aroma, colour intensity and fragrance of the finished product [1,29,35,36,37,38]; refer to Supplementary Table S1 for more detail.

In our previous study, recombinant *Pediococcus acidilactici* BD16 (*alaD^+^*) was shown to contribute to development of several flavour enhancers (such as acetaldehyde, alaninol, aminopentols, benzene methanol, 3-butynol, octanoic acid, ribitol) and therapeutic compounds (such as actinobolin, adamantanemethylamine, aminononadecane, amphetamine, 8-azanonane, benzeneethanamine, guanosine, heptadecanenitrile, 86isopropyluriedoacetate, nortriptyline) and rimantadine for the value addition of ginger, ki87wi, plum and rose wines [17]. Additionally, a proof-of-concept study was conducted in our laboratory to evaluate the role of *Pediococcus acidilactici* BD16 (*alaD*^+^) in developing functional buttermilk and soymilk drinks. Metabolomic analysis using GC-MS technique has revealed the enhancement of secondary metabolites (such as acetic acid, 2-aminononadecane, azanonane, benzaldehyde, 1,2-benzenedicarboxylic acid, benzoic acid, chloroacetic acid, colchicine, 1-dodecene, heptadecanenitrile, hexadecanal, 3-octadecene, 4-octen-3-one, quercetin and triacontane), which improved health promoting attributes of the fermented drinks. In addition to this, LAB fermented drinks have considerably reduced bitterness, rancidity and unpleasant odour due to reduction in the levels of 2-bromopropionic acid, formic acid, 8-heptadecene, 1-pentadecene and propionic acid [15].

## 4. Discussion and Conclusions

Fermentation is considered a vital tool for expanding nutritive, functional and sensory traits of beverages [39]. In recent years, there has been an increased inclination of consumers towards healthy, nutritive and functional beverages, which presumably are the optimal vehicles to transport nutrients and bioactive compounds into the body. Fermented beverages, especially, facilitate enhanced bioavailability of phytoconstituents such as anthocyanins, carotenoids, dietary fibres, fatty acids, flavonoids, minerals, phenolic derivatives, vitamins, and delivery of probiotics through dietary supplementation. The consumption of health-promoting dietary supplements can be helpful in establishing a parallel line of defence against important human diseases, particularly in their early stages of development [40]. It has been suggested that the daily intake of wheatgrass juice improves blood flow and aids digestion and general detoxification of the body due to the presence of anti-oxidative bioflavonoids such as apigenin, quercitin, luteoline and minerals in it [41].

In a study, wheatgrass juice was utilized for the value addition of kombucha—a traditional fermented black tea drink. Sweetened black tea and wheatgrass juice were mixed in different ratios and fermented using microbial consortium consisting of a yeast strain, namely, *Dekkera bruxellensis*, and two strains of acetic acid bacteria viz. *Gluconacetobacter rhaeticus* and *Gluconobacter roseus* at 29 ± 1 °C for 12 days. The fermented drinks have higher total phenol and flavonoid content, which also elevated their antioxidant activity as compared to traditional kombucha. The beneficial effects of black tea were enhanced after mixing with wheatgrass juice as compared to traditional kombucha because of elevated amounts of caffeic acid, catechin, chlorogenic acid, ferulic acid, gallic acid, rutin etc. as compared to traditional ones. The highest antioxidant activity was observed when black tea and wheatgrass juice were mixed in equal proportions and fermented for three days [42]. In the present study, improved biochemical and phenolic profiles of fermented wheatgrass juice were observed after two-stage fermentation using *Saccharomyces cerevisiae* and *P*. *acidilactici* BD16 (*alaD*^+^). Additionally, the use of *Saccharomyces cerevisiae* contributed to bioconversion of coumaric acid, hydroxybenzoic acid, hydroxycinnamic acid, quinic acid, etc. into respective derivatives; glycosylation and prenylation of flavonoids; glycosylation of lignans; sulphonation of phenols; synthesis of carotenoids, diarylnonanoids, flavanones, stilbenes, steroids, quinolones, di- and tri-terpenoids; and tannin in the fermented wheatgrass juice. The study also displays the role of *P*. *acidilactici* BD16 (*alaD*^+^) in expanding the functional profile of fermented wheatgrass juice by synthesizing anthraquinone, sterols and triterpene, in addition to glycosylation of flavonoids and lignins, and derivatization of benzoic acid, hydroxycoumaric acid and quinic acid. The present study also provides sufficient biochemical basis to support previously acclaimed therapeutic benefits of freeze-dried wheatgrass powder, fermented wheatgerm extracts, etc. mentioned in the scientific literature (as discussed in the Introduction). In lieu of the results described above, it can be established that the utilization of two-stage fermentation process improves nutritive as well as functional profile of the fermented wheatgrass juice. However, dedicated in vitro and in vivo investigations of the fermented wheatgrass juice need to be conducted for further validation of its nutritive, functional and therapeutic benefits. Moreover, fermented wheatgrass juice can be used to supplement traditional beverages to develop dietary supplements with health-promoting attributes.

## Figures and Tables

**Table 1:** Biochemical properties of fermented wheatgrass juice.

Parameters	Wheatgrass Juice	After Primary Fermentation	After Secondary Fermentation
Total solid content (%)	22.9	6.3	6.2
Moisture content (%)	77.1	93.7	93.7
Total acids (%)	0.2 ± 0.01	1.08 ± 0.66	0.65 ± 0.06
Ethanol content (%)	0.0	3.4 ± 0.004	3.7 ± 0.005
Total proteins (mg/mL)	4.6 ± 0.10	4.07 ± 0.32	4.52 ± 0.30
Total phenols (mg GAE/mL)	0.41 ± 0.01	1.45 ± 0.04	1.36 ± 0.03
Total flavonoids (mg QE/L)	0.39 ± 0.010	0.17 ± 0.011	0.169 ± 0.015
Total anthocyanins (mg Cy3G/L)	0.97 ± 0.16	3.50 ± 0.67	4.53 ± 0.05
Total carotenoids (mg/L)	0.25 ± 0.017	0.31 ± 0.014	0.31 ± 0.09
Beta-carotenes (mg/L)	0.35 ± 0.01	0.71 ± 0.019	0.73 ± 0.021
Lycopenes (mg/L)	0.28 ± 0.003	0.33 ± 0.017	0.34 ± 0.015
Tint of wine	2.757 ± 0.023	0.020 ± 0.003	0.110 ± 0.016
Brilliance of wine (%)	6.6 ± 0.057	42.2 ± 0.121	55.0 ± 0.551

Values are presented as mean ± standard deviation of triplicates.

**Table 2:** Characteristics of secondary metabolites identified in fermented wheatgrass juice by LC-MS-MALDI-TOF/TOF.

Sr. No.	Secondary Metabolites	Class	WGJ	WGJ-PF	WGJ-SF
1	1-(2,6-Dihydroxyphenyl)-9-(4-hydroxy-3-methoxyphenyl)	Phenol derivative	+	−	−
2	1-O-Caffeoyl-β-D-glucose	Hydroxycinnamic acid glycoside	−	+ *	−
3	1-O-Sinapoyl-β-D-glucose	Hydroxycinnamic acid glycoside	−	+ *	−
4	2-Methyl-4,6-dinitrophenol	Phenol derivative	+	−	+ ^
5	2,4-Dimethylphenol	Phenol derivative	−	−	+ ^
6	3-Caffeoyl quinic acid	Quinic acid & derivatives	−	+ *	+
7	3-or 4-hydroxyphenyl propionic acid sulphate	Hydroxymono-carboxylic acid	+	−	−
8	3-p-Coumaroyl quinic acid	Quinic acid & derivatives	−	+ *	−
9	6-C-hexosyl-chrysoeriol-O-rhamnoside-O-hexoside	Flavonoid	−	+ *	−
10	8-Prenyl naringenin	Prenylflavonoid	−	+ *	−
11	β-Amyrin	Triterpenoid	−	+ *	−
12	Ampelosin D	Stilbenes	+	+	−
13	Apigenin-6-O-glucoside	Flavonoid glucoside	+	−	−
14	Apigenin-7-O-glucoside	Flavonoid glucoside	+	−	+ ^
15	Austrabailignan-7	Lignan	+	+	+
16	Avenasterol	Stigmastane	+	−	+ ^
17	Betanin (red pigment)	Tyrosine derivative	−	+ *	−
18	5-Campestenone	Sterol	+	+	−
19	Carnosic acid	Diterpenoid	+	−	−
20	Catechin	Flavonoid	−	+ *	−
21	Catechin gallate	Flavans	+	−	−
22	Chrysoeriol-C-hexoside-C-pentoside	Flavonoid glucoside	−	+ *	−
23	Chlorogenic acid	Quinic acid & derivatives	−	+ *	+
24	Cinnamic acid	Cinnamic acid	+	−	−
25	Cirsiliol	Flavonoid derivative	+	+	−
26	Deoxyschisandrin (pigment)	Tannin	−	+ ×	−
27	Delphinidin-3-glucoside	Polyphenol	+	+	+
28	Dihydrocaffeic acid-3-O-glucuronide	Phenolic glycosides	−	+ *	−
29	Dihydroferulic acid sulphate	Phenyl sulphates	−	+ *	−
30	Dihydroguaiaretic acid	Lignan	+	−	−
31	Docosenoic acid	Unsat. fatty acid derivative	+	+	+
32	Ellagic acid	Polyphenol	−	+ *	−
33	Epicatechin	Flavonoid	+	−	−
34	Epicatechin gallate	Flavonoid	+	−	−
35	Esculin	Coumarin glucoside	−	+ *	−
36	Eugenol	Allylbenzene	+	−	−
37	Ferulic acid	Hydroxycinnamic acid	+	+	+
38	Fragransin D1	Lignan	−	−	+ ^
39	Fraxetin-7-O-sulfate	Hydroxycoumarin derivative	+	−	+ ^
40	Fucosterol	Sterol	+	−	+ ^
41	Gibberellin acid 8-hexose-gibberellin	Diterpenoid	−	+ *	+
42	Gallic acid	Phenolic acid	+	−	−
43	Gallic acid 4-O-glucoside	Phenolic acid derivative	+	−	−
44	2-Hydroxy-4-methoxy-3,6-dimethylbenzoic acid	Benzoic acid derivative	+	−	+ ^
45	Kaempferol-rha-xyl-gal	Flavonoid glucoside	−	+ *	−
46	Laricitrin-3-O-rutinose	Flavonoid glucoside	−	+ *	−
47	Linoleic acid isomer 1or 2	Unsat. fatty acid	−	+ *	−
48	Luteolin-8-C-glucoside	Flavonoid	−	−	+ ^
49	Malabaricone B	Diarylnonanoids	−	+ *	−
50	Malvidin-3-(6-O-acetyl)glucoside	Flavonoid glucoside	+	−	−
51	Malvidin-3-O-glucoside-4-vinylphenol	Flavonoid glucoside	+	−	−
52	Malvidin-3-O-rutinoside	Flavonoid glucoside	−	+ *	+
53	Monotropein	Monoterpenoid	+	−	−
54	Malvidin-3-glucoside-4-vinyl(epi) catechin (pigment)	Flavanol-anthocyanin adduct	−	−	+ ^
55	Myricetin	Flavonoid	+	+	−
56	Myricetin-3-O-glucoside	Flavonoid glucoside	+	+	+
57	Myricetin-3-O-rhamnoside	Flavonoid glucoside	−	−	+ ^
58	Myricetin-7-O-pentoside	Flavonoid glucoside	−	−	+ ^
59	Naringenin glucuronide sulfate	Flavanone glucuronide	−	+ *	+
60	Naringenin sulfate	Flavanone	−	+ *	−
61	Neotigogenin acetate	Triterpenoid	−	−	+ ^
62	Neoeriocitrin	Flavanone	−	+ *	−
63	Pallidol (Resveratrol dimer)	Stilbenoid	+	−	−
64	p-Coumaroyl-hexose-methylglutarate	Hydroxycinnamic acid derivative	−	+ *	+
65	Pelarogonidin (pigment)	Flavonoid	+	+	−
66	Pentacosanoic acid	Sat. Fatty acid	−	+ *	−
67	Pentahydroxydimethoxy flavone	Flavonoid	−	+ *	−
68	Peonidin	Flavonoid	+	+	−
69	Peonidin-3-O-glucoside (pigment)	Flavonoid glucoside	+	+	−
70	Peonidin-3-O-rutinoside-5-glucoside (pigment)	Flavonoid glucoside	+	+	−
71	Peonidin-3-O-sambioside-5-O-glucoside (pigment)	Flavonoid glucoside	−	−	+ ^
72	Peonidin-3-O-rutinoside (pigment)	Flavonoid glucoside	+	−	−
73	Pentahydroxytrimethoxyflavones	Flavonoid	+	+	−
74	Petunidin-3-O-glucoside	Flavonoid glucoside	−	+ *	−
75	Phlorizin	Flavonoid glucoside	+	+	−
76	Phloretin-3’, 5’-di-C-β-glucoside	Diarylpropanoid	+	+	+
77	Pinobanksin arabinose	Lignan glycoside	−	+ *	−
78	Pinocembrin-O-arabirosyl-glucoside	Flavonoid glycoside	−	+ *	−
79	Piperyline	Alkaloid	+	−	−
80	Procyanidin B1	Flavonoid	+	−	−
81	Procyanidin B2	Flavonoid	+	−	−
82	Procyanidin dimer gallate	Flavonoid	+	+	−
83	Prodelphinidin A-type	Flavonoid	+	−	−
84	Protocatechuic acid-4-O-glucoside	Hydroxybenzoic acid derivative	−	+ *	−
85	Quercetin	Flavonoid	+	+	+
86	Quercetin-7-O-malonynyl-hexoside	Flavonoid glucoside	−	+ *	−
87	Quinic acid	Quinic acid	−	+ *	−
88	Riboflavin	Vitamin B2	+	+	−
89	Rhein	Anthraquinone	−	−	+ ^
90	Salviaflaside derivative	Phenylpropanoid	−	+ *	−
91	Salvianolic acid B isomer 1or 2	Flavonoid	−	+ *	−
92	Spermidine-N1,10-di-caffeicacid-N5-p-coumaric acid	Polyamine-quinic acid adduct	−	−	+ ^
93	Spermidine-N5,10-di-p-coumaric acid-N1-caffeic acid	Polyamine-quinic acid adduct	−	−	+ ^
94	Syringetin	Flavonoid	+	−	-
95	Syringetin 3-O-hexoside	Flavonoid glucoside	+	+	+
96	Taxifolin	Flavonoid	+	+	-
97	Taxifolin-O-pentoside	Flavonoid glucoside	+	+	+
98	Taxifolin-3-O-glucoside	Flavonoid glucoside	−	+ *	−
99	Taxifolin-3-O-rhamnoside	Flavonoid glucoside	+	+	+
100	trans-Scirpusin A	Stilbene	−	+ *	−
101	Tricin	Flavonoid	+	-	−
102	Trihydroxy-ent-kauranoic acid	Diterpene derivative	−	+ *	−
103	Viniferal	Hydroxystilbenoid	+	−	−
104	α-Viniferin	Stilbene	+	+	−
105	Vanillic acid 4-sulfate	Hydroxybenzoic acid derivative	−	+ *	+
106	Violanone	Flavonoid	−	+ *	−
107	Vebonol	Steroid	−	+ *	+
108	Zeaxanthin (pigment)	Carotenoid	−	+ *	−

WGJ—wheatgrass juice; WGJ-PF—wheatgrass juice after primary fermentation; WGJ-SF—wheatgrass juice after secondary fermentation; present (+), absent (−); * phenolics contributed by yeast; ^ phenolics contributed by *P. acidilactici* BD16 (*alaD*+).

## Data Availability

Data is contained within the article or Supplementary Table S1.

## Supplementary Materials

The following supporting information can be downloaded at: https://www.mdpi.com/article/10.3390/foods12081624/s1, Table S1: Detection of secondary metabolites in fermented wheatgrass juice using LC-MS-MALDI-TOF/TOF and their characteristics [29,35,36,37,38,43,44,45,46,47,48,49,50,51,52,53,54,55,56,57,58,59,60,61,62,63,64,65,66,67,68,69,70,71,72,73,74,75,76,77,78,79,80,81,82,83,84,85,86,87,88,89,90,91,92,93,94,95,96,97,98,99,100,101,102,103,104,105,106,107,108,109,110,111,112,113,114,115,116,117,118,119].
