# Supplementary material for: Phenolic Biotransformations in Wheatgrass Juice after Primary and Secondary Fermentation
Source: Foods. 2023 Apr 12;12(8):1624. doi: 10.3390/foods12081624 (PMC10138189; doi:10.3390/foods12081624)
Supplement: Supplementary file 1 [file foods-12-01624-s001.zip › foods-2238825-supplementary.pdf]

Table S1. Detection of secondary metabolites in fermented wheatgrass juice using LC-MS-MALDI-TOF/TOF and their characteristics

RT	Observed m/z value	Metabolite detected	Identity of compound	Activities	References (Compound identity)	References (Biological property)
1.4	122.9	M+H	2,4- Dimethylphenol	Anti-oxidant	[1]	[2]
30.6	149.0	M+H	Cinnamic acid	Anti-cancer<br>Anti-diabetic<br>Anti-microbial<br>Cardioprotective<br>Hepatoprotective<br>Neuroprotective	[3]	[4]
1.3	170.9	M-H	Gallic acid	Anti-cancer<br>Anti-oxidant	[5]	[6]
1.3	182.9	$M+NH_4$	Eugenol	Anti-inflammatory	[7]	[7]
27.0	191.0	M-H	Quinic acid	Anti-diabetic<br>Anti-inflammatory<br>Anti-oxidant	[8]	[9]
1.4	198.9	Fragment<br>M+3H	2-Methyl 4,6- dinitrophenol,<br>Hydroxymethoxydimethyl benzoic acid	Anti-oxidant<br>Anti-inflammatory	[1,10]	[11]
30.6	245.0	M-H<br><br>Fragment<br>Fragment<br>Fragment<br>Fragment<br>Fragment<br>Fragment<br>Fragment	3-or 4-Hydroxyphenyl propionic acid sulphate,<br>Catechin,<br>Catechin gallate,<br>Epicatechin,<br>Epicatechin gallate,<br>Procyanidin B1,<br>Procyanidin B2,<br>Prodelphinidin A	Anti-cancer<br>Anti-diabetic<br>Anti-inflammatory<br>Anti-oxidant<br>Anti-tumorigenic	[12,13]	[14]
19.0	246.2	M-H	Vanillic acid 4-sulfate	Anti-inflammatory<br>Anti-oxidatnt<br>Neuroprotective	[15]	[16]
1.4	257.1	Fragment	Ellagic acid	Anti-oxidant	[17]	[18]
33.3	271.2	Fragment	Pinobanksin arabinose	Anti-bacterial<br>Anti-inflammatory<br>Anti-oxidant<br>Anti-parasitic<br>Anti-proliferative<br>Immunomodulatory	[19]	[20]
33.3	272.2	M+H	Piperyline	Anti-cancerous<br>Anti-tumorigenic	[21]	[21]
21.8	274.2	M+3H	Pelarogonidin	Anti-oxidant<br>Anti- cancerous	[22]	[22]
21.8	275.2	M-H	Dihydroxyferulic acid sulphate	Anti-cancer<br>Anti-diabetic<br>Anti-inflammatory<br>Anti-microbial<br>Anti-oxidant	[12]	[23]
1.6	277.0	M+H	Linoleic acid isomer 1 or 2	Anti-atherogenic<br>Anti-cancer	[24]	[25]
34.6	282.2	M+2H	Rhein	Anti-cancer<br>Anti-inflammatory<br>Anti-microbial<br>Anti-oxidant	[26]	[27]

1.4	288.9	M+H	Fraxetin-7-O-sulfate	Anti-bacterial<br>Anti-osteoporosis<br>Anti-oxidant	[10]	[28]
18.9	291.2	M+H	Catechin	Anti-inflammatory<br>Growth inhibitory	[10]	[14]
30.6	301.1	Fragment<br>M-3H	Delphinidin-3-glucoside,<br>Quercetin	Anti-bacterial<br>Anti-inflammatory<br>Anti-oxidant<br>Anti-viral<br>Gastroprotective<br>Immunomodulatory	[13]	[29]
30.6	302.2	M+H<br>Fragment<br>Fragment<br><br>Fragment<br><br>Fragment	Peonidin,<br>Peonidin-3-O-glucoside,<br>Peonidin-3-O-rutinoside-5-glucoside,<br>Peonidin-3-O-sambioside-5-O-glucoside,<br>Peonidin-3-O-rutinoside	Anti-bacterial<br>Anti-inflammatory<br>Anti-oxidant<br>Anti-viral<br>Gastroprotective<br>Immunomodulatory	[13,10]	[29]
30.6	303.3	Fragment<br>M-H<br>Fragment<br>Fragment	Myricetin-3-O-glucoside,<br>Taxifolin,<br>Taxifolin-O-pentoside,<br>Taxifolin-3-O-rhamnoside	Anti-bacterial<br>Anti-inflammatory<br>Anti-oxidant<br>Anti-viral<br>Gastroprotective<br>Immunomodulatory	[13]	[29]
26.4	315.1	Fragment<br><br>M-H<br><br>Fragment<br>M-H	Malvidin-3-(6-O-acetyl) glucoside,<br>Protocatechuic acid 4-O-glucoside,<br>Petunidin-3-O-glucoside,<br>Violanone	Anti-ageing<br>Anti-asthmatic<br>Anti-athrogenic<br>Anti-bacterial<br>Anti-cancer<br>Anti-diabetic<br>Anti-inflammatory<br>Anti-oxidant<br>Anti-tumorigenic<br>Anti-ulcer<br>Neuroprotective	[13,30]	[31]
27.0	319.0	M+H<br>Fragment	Myricetin,<br>Riboflavin	Anti-cancer<br>Anti-oxidant<br>Anti-viral	[3]	[22]
26.0	330.3	Fragment	Syringetin 3-O-hexoside	Anti-bacterial<br>Anti-inflammatory<br>Anti-oxidant	[32]	[33]
26.0	331.3	M+H<br>M+H<br>Fragment<br>Fragment<br>M-H<br>M-H	Cirsiliol,<br>Tricin,<br>Malvidin-3-rutinoside,<br>Malvidin-acetyl-glucoside,<br>Carnosic acid,<br>Gallic acid 4-O-glucoside	Anti-inflammatory<br>Anti-neoplastic<br>Anti-oxidant<br>Cardioprotective<br>Neuroprotective	[10,34,35]	[6,22]
26.4	337.1	M-H	3-p-Coumaroylquinic acid	Anti-inflammatory<br>Anti-microbial<br>Cardioprotective	[36]	[37]
35.1	338.3	Fragment	Ferulic acid	Anti-cancer<br>Anti-diabetic<br>Anti-inflammatory<br>Anti-microbial<br>Anti-oxidant	[38]	[23]

37.5	339.3	M-H<br>M+H	8-Prenylnaringenin,<br>Docosenoic acid	Anti-cancer<br>Anti- inflammatory<br>Anti-microbial<br>Anti-oxidant<br>Anti-viral	[10,39]	[40,41]
27.0	341.0	M-H<br>Fragment<br>M+H<br>M+H	1-O-Caffeoyl glucose,<br>1-O-Sinapoyl-β-D-glucose,<br>8-Prenylnaringenin,<br>Esculin	Anti-cancer<br>Anti-inflammatory<br>Anti-oxidant<br>Anti-viral	[13,36]	[42]
32.4	343.1	M+H	Malabaricone B	Anti-inflammatory<br>Anti-microbial	[7]	[7]
23.9	346.3	Fragment	Pallidol	Anti-microbial<br>Anti-oxidant	[13]	[43]
1.3	347.0	M+H	Syringetin	Anti-oxidant	[22]	[44]
1.3	348.0	$M+NH_4$	Dihydroguaiaretic acid	Anti-oxidant	[7]	[7]
18.9	351.2	M-H<br><br>M-H	Trihydroxy-ent-kauranoic acid,<br>Naringenin sulfate	Anti-bacterial<br>Anti-cancer<br>Anti-convulsant<br>Anti-inflammatory<br>Anti-leishmania<br>Anti-mutagenic	[45,46]	[47,48]
19.0	353.2	M-H<br>M-H	3-Caffeoylquinic acid,<br>Chlorogenic acid	Anti-bacterial<br>Anti-helminthic	[15,49]	[50]
33.7	357.2	M-H	Dihydrocaffeic acid 3-O-glucuronide	Anti-oxidant	[15]	[51]
27.0	358.2	Fragment	Avenasterol	Anti-diabetic<br>Anti-inflammatory<br>Cardioprotective	[10]	[52]
27.0	360.2	$M+NH_4$	Austrobailignan-7	Anti-inflammatory<br>Anti-oxidant<br>Anti-tumorigenic	[7]	[7]
1.6	365.1	M+Na<br>Fragment	Austrobailignan-7,<br>Phloretin-3’,5-di-C-β-glucoside	Anti-inflammatory<br>Anti-oxidant<br>Anti-tumorigenic<br>Anti-viral<br>Immunosuppressive<br>Cardioprotective	[7,53]	[7]
25.4	375.3	Fragment	Pentahydroxytrimethoxy flavone	Anti-angiogenic<br>Anti-fungal<br>Anti-tumorigenic<br>Anti-viral<br>Immunomodulatory	[10]	[54]
1.6	381.0	M+H	Pentacosenoic acid	Anti-microbial<br>Anti- thrombotic	[10]	[55]
1.3	391.1	M+H	Monotropein	Anti-apoptotic<br>Anti-catabolic<br>Anti-fungal<br>Anti-inflammatory<br>Anti-nociceptive<br>Anti-oxidant	[7]	[7]
34.6	395.3	M+Na<br><br><br>Fragment	1-(2,6-Dihydroxypheny l)-9-(4-hydroxy-3-methoxy phenyl),<br>Catechin gallate	Anti-angiogenic<br>Anti-melanogenic<br>Anti-oxidant<br>Anti-plasmodic	[7,13]	[56]
34.6	399.2	M+H	Campestenone	Anti-cancerous<br>Anti-diabetic<br>Anti-inflammatory	[10]	[57]

35.1	406.3	M+NH_4<br>Fragment	Fragransin D1,<br>Pallidol	Anti-microbial<br>Anti-oxidant	[7,13]	[43]
30.9	413.2	M+H<br>M+H<br>Fragment	Avenasterol,<br>Fucosterol,<br>Apigenin-7-O-glucoside	Anti-diabetic<br>Anti-inflammatory<br>Cardioprotective	[10,13]	[52]
28.5	414.2	Fragment	Malvidin 3-O-glucoside-4-vinylphenol	Anti-ageing<br>Anti-asthmatic<br>Anti-atherogenic<br>Anti-bacterial<br>Anti-cancer<br>Anti-diabetic<br>Anti-inflammatory<br>Anti-oxidant<br>Anti-tumorigenic<br>Anti-ulcer<br>Neuroprotective	[32]	[31]
24.4	415.2	M-H	Deoxyschisandrin	Anti-cancerous<br>Anti-inflammatory	[30]	[58]
33.7	427.2	M+H<br>Fragment	β-Amyrin,<br>trans-Scirpusin A	Anti-microbial<br>Anti-obesity<br>Anti-parasitic<br>Anti-tumorigenic	[10,13]	[59,60]
33.7	429.2	Fragment	Pinocembrin-O-arabirosyl-glucoside	Anti-cancer<br>Anti-inflammatory<br>Anti-microbial<br>Anti-oxidant	[61]	[62]
34.6	431.1	M-H	Apigenin 6-C-glucoside	Anti-inflammatory<br>Anti-mutagenic<br>Anti-oxidant<br>Anti-viral	[15]	[52]
24.4	437.1	M+H<br>Fragment<br>Fragment<br>Fragment	Phlorizin,<br>Ampelosin D,<br>α-Viniferin,<br>Procyanidin dimer gallate	Anti-inflammatory<br>Anti-microbial<br>Anti-oxidant	[10,13]	[63]
38.8	449.7	M-H<br>M+H	Myricetin 7-O-pentoside,<br>Luteolin-8-C-glucoside	Anti-cancer<br>Anti-inflammatory<br>Anti-osteoporosis<br>Anti-oxidant<br>Anti-thrombotic<br>Cardioprotective	[10,32]	[64]
38.8	453.1	M-H<br><br>M+H	p-Coumaroyl-hexoside-methylglutarate,<br>Vebonol	Anti-cancer<br>Anti-diabetic<br>Anti-inflammatory<br>Anti-oxidant	[10,65]	[66]
1.4	458.8	M-H	Neotigogenin acetate	Anti-cancer	[5]	[67]
30.0	463.3	Fragment<br>M-H	Peonidin-3-O-rutinoside,<br>Myricetin 3-O-rhamnoside	Anti-Alzheimer's<br>Anti-inflammatory<br>Anti-Parkinson's	[10,30]	[68,69]
24.4	467.1	M+H	Taxifolin-3-O-glucoside	Anti-oxidant	[10]	[70]
33.7	521.3	M-H	Salviaflaside derivatives	Anti-inflammatory	[46]	[71]
1.6	527.1	M+H<br><br>M-H	Gibberellin acid 8-hexose-gibberellin,<br>Naringenin glucuronide sulfate	Anti-oxidant	[10,45]	[72]
18.9	549.1	M-H	Quercetin-7-O-malonylhexoside	Anti-angiogenic<br>Anti-inflammatory<br>Anti-oxidant<br>Anti-tumorigenic	[65]	[73]

30.0	551.3	M+H	Quercetin 3-O-(6'-malonyl-glucoside),<br>Betanin	Anti-angiogenic<br>Anti-inflammatory<br>Anti-oxidant<br>Anti-tumorigenic	[30]	[73]
33.7	569.5	M+H	Zeaxanthin	Anti-oxidant<br>Anti-inflammatory	[10]	[74]
34.6	573.4	M-H	Viniferal	Neuroprotective	[13]	[75]
30.0	595.3	M-H<br>M+H	Neoeriocitrin,<br>Chrysoeriol C-hexoside-C-pentoside	Anti-oxidant<br>Anti-inflammatory	[10,30]	[61]
1.4	598.8	M-H	Spermidine- N5,10-di-p-coumaric acid-N1-caffeic acid	Anti-fungal<br>Anti-oxidant	[19]	[76]
30.9	614.3	M-H	Spermidine- N1,10-di-caffeic acid-N5-p-coumaric acid	Anti-fungal<br>Anti-oxidant	[19]	[76]
30.0	639.4	M-H	Laricitrin-3-O-rutinose	Anti-allergic<br>Anti-inflammatory<br>Anti-microbial<br>Anti-oxidant	[8]	[77]
30.0	640.4	M+H	Malvidin 3-O-rutinoside	Anti-inflammatory<br>Anti-oxidant	[10]	[22]
1.4	717.9	M+2H	Salvianolic acid B isomer 1 or 2	Anti-cancer<br>Anti-inflammatory<br>Anti-oxidant<br>Cardioprotective	[24]	[28]
30.0	727.4	Fragment	Kaempferol-rha-xyl-gal	Anti-oxidant	[78]	[79]
1.4	758.7	M+H	Peonidin 3-O-sambioside-5-O-glucoside	Anti-oxidant<br>Anti-tumorigenic<br>Cardioprotective	[30]	[80]
34.6	771.4	M+H	6-C-hexosyl-chrysoeriol-O-rhamnoside-O-hexoside	Anti-cancer<br>Anti-inflammatory<br>Anti-microbial<br>Anti-osteoporosis<br>Anti-insect<br>Neuroprotective	[10]	[81]
30.9	805.5	Fragment	Malvidin-3-glucoside-4-vinyl (epi) catechin	Anti-inflammatory<br>Anti-oxidant	[82]	[22]

RT- Retention time

References:

1. Nakamura, S.; Takino, M.; Daishima, S. Trace Level Determination of Phenols as Pentafluorobenzyl Derivatives by Gas Chromatography - Negative-Ion Chemical Ionization Mass Spectrometry. *Analyst* **2001**, *126* (6), 835–839.

2.Chukicheva, I. Yu.; Krylova, M. V.; Buravlev, E. V.; Suponitskii, K. Yu.; Kutchin, A. V. Alkylation of 2,4-Dimethylphenol with (+)-α- and (−)-β-Pinenes in the Presence of Aluminum Xylenolate. *Russian J. Struct. Chem.* **2014**, *50* (4), 589–595.

3. Pamplona, S.; Sá, P.; Lopes, D.; Costa, E.; Yamada, E.; Silva, C.E.; Arruda, M.; Souza, J.; Da Silva, M. *In Vitro* Cytoprotective Effects and Antioxidant Capacity of Phenolic Compounds from the Leaves of *Swietenia macrophylla*. *Molecules* **2015**, *20*, 18777-18788.

4. Rychlicka, M.; Rot, A.; Gliszczyńska, A. Biological Properties, Health Benefits and Enzymatic Modifications of Dietary Methoxylated Derivatives of Cinnamic Acid. *Foods* **2021**, *10* (6), 1417.

5. Tlhapi, D.B.; Ramaite, I.D.I.; Anokwuru, C.P. Metabolomic Profiling and Antioxidant Activities of *Breonadia salicina* Using ^{1}H-NMR and UPLC-QTOF-MS Analysis. *Molecules* **2021**, *26*, 6707.

6. Kahkeshani, N.; Farzaei, F.; Fotouhi, M.; Alavi, S.S.; Bahramsoltani, R.; Naseri, R.; Momtaz, S.; Abbasabadi, Z.; Rahimi, R.; Farzaei, M.H.; Bishayee, A. Pharmacological effects of gallic acid in health and diseases: A mechanistic review. *Iranian J. Basic Med. Sci.* **2019,** *22*(3), 225.

7. Marulasiddaswamy, K.M.; Nuthan, B.R.; Channarayapatna Ramesh, S.; Bajpe, S.N.; Kumara, K.K.S.; Sekhar, S.K.K. HRLC-MS based Profiling of Phytochemicals from Methanol Extracts of Leaves and Bark of *Myristica dactyloides* Gaertn. from Western Ghats of Karnataka, India. *J. Appl. Biol. Biotechnol.* **2021,** *9*(05), 124-135.

8. Lyubchyk, S.; Shapovalova, O.; Lygina, O.; Oliveira, M. C.; Appazov, N.; Lyubchyk, A.; Charmier, A. J.; Lyubchik, S.; Pombeiro, A. J. L. Integrated Green Chemical Approach to the Medicinal Plant Carpobrotus Edulis Processing. *Sci. Rep.* **2019**, *9* (1), 1-12.

9. Mortelé, O.; Jörissen, J.; Spacova, I.; Lebeer, S.; van Nuijs, A. L. N.; Hermans, N. Demonstrating the Involvement of an Active Efflux Mechanism in the Intestinal Absorption of Chlorogenic Acid and Quinic Acid Using a Caco-2 Bidirectional Permeability Assay. *Food Funct.* **2021**, *12* (1), 417–425.

10. Razgonova, M.P.; Zakharenko, A.M.; Gordeeva, E.I.; Shoeva, O.Y.; Antonova, E.V.; Pikula, K.S.; Koval, L.A.; Khlestkina, E.K.; Golokhvast, K.S. Phytochemical Analysis of Phenolics, Sterols, and Terpenes in Colored Wheat Grains by Liquid Chromatography with Tandem Mass Spectrometry. *Molecules* **2021**, *26*, 5580.

11. Minatel, I. O.; Borges, C. V.; Ferreira, M. I.; Gomez, H. A. G.; Chen, C.-Y. O.; Lima, G. P. P. Phenolic Compounds: Functional Properties, Impact of Processing and Bioavailability. *Phenolic Comp. Biol. Act.* **2017**, 8, 1-24.

12. Pekkinen, J.; Rosa, N. N.; Savolainen, O.-I.; Keski-Rahkonen, P.; Mykkänen, H.; Poutanen, K.; Micard, V.; Hanhineva, K. Disintegration of Wheat Aleurone Structure Has an Impact on the Bioavailability of Phenolic Compounds and Other Phytochemicals as Evidenced by Altered Urinary Metabolite Profile of Diet-Induced Obese Mice. *Nutr. Metab.* **2014**, *11* (1), 1.

13. Goufo, P.; Singh, R.K.; Cortez, I. A Reference List of Phenolic Compounds (Including Stilbenes) in Grapevine (*Vitis vinifera* L.) Roots, Woods, Canes, Stems, and Leaves. *Antioxidants* **2020**, *9(5)*, 398.

14. Rauf, A.; Imran, M.; Abu-Izneid, T.; Iahtisham-Ul-Haq; Patel, S.; Pan, X.; Naz, S.; Sanches Silva, A.; Saeed, F.; Rasul Suleria, H. A. Proanthocyanidins: A Comprehensive Review. *Biomedicine & Pharmacotherapy* **2019**, *116*, 108999.

15. Hameed, A.; Liu, Z.; Wu, H.; Zhong, B.; Ciborowski, M.; Suleria, H.A.R. A Comparative and Comprehensive Characterization of Polyphenols of Selected Fruits from the Rosaceae Family. *Metabolites* **2022**, *12*, 271.

16. Ingole, A.; Kadam, M.; Dalu, A. P.; Kute, S. M.; Mange, P. R.; Theng, V. D.; Lahane, O. R.; Nikas, A. P.; Kawal, Y. V.; Nagrik, S. U.; Patil, P. A. A Review of the Pharmacological Characteristics of Vanillic Acid. *J. Drug Deliv. Ther.* **2021**, *11*, 200-204.

17. Wyrepkowski, C.C.; Gomes da Costa, D.L.M.; Sinhorin, A.P.; Vilegas, W.; De Grandis, R.A.; Resende, F.A.; Varanda, E.A.; Dos Santos, L.C. Characterization and Quantification of the Compounds of the Ethanolic Extract from *Caesalpinia ferrea* Stem Bark and Evaluation of Their Mutagenic Activity. *Molecules* **2014**, *19*, 16039-16057.

18. Kim, J.-P.; Lee, I.-K.; Yun, B.-S.; Chung, S.-H.; Shim, G.-S.; Koshino, H.; Yoo, I.-D. Ellagic Acid Rhamnosides from the Stem Bark of Eucalyptus Globulus. *Phytochemistry* **2001**, *57* (4), 587–591.

19. Gardana, C.; Del Bo', C.; Quicazán, M. C.; Corrrea, A. R.; Simonetti, P. Nutrients, Phytochemicals and Botanical Origin of Commercial Bee Pollen from Different Geographical Areas. *J. Food Compos. Anal.* **2018**, *73*, 29–38.

20. Alday, E.; Valencia, D.; Carreño, A. L.; Picerno, P.; Piccinelli, A. L.; Rastrelli, L.; Robles-Zepeda, R.; Hernandez, J.; Velazquez, C. Apoptotic Induction by Pinobanksin and Some of

Its Ester Derivatives from Sonoran Propolis in a B-Cell Lymphoma Cell Line. *Chem.-Biol. Interact.* **2015**, *242*, 35–44.

21. Olalere, O. A.; Abdurahman, N. H.; Yunus, R. bin M.; Alara, O. R.; Kabbashi, N. A. Chemical Fingerprinting of Biologically Active Compounds and Morphological Transformation during Microwave Reflux Extraction of Black Pepper. *Chem. Data Collect.* **2018**, *17-18*, 339–344.

22. Kaur, B.; Kumar, B.; Kaur, G.; Chakraborty, D.; Kaur, K. Application of Recombinant Pediococcus Acidilactici BD16 (Fcs + /Ech +) in Malolactic Fermentation. *Appl. Microbiol. Biotechnol.* **2015**, *99* (7), 3015–3028.

23. Zduńska, K.; Dana, A.; Kolodziejczak, A.; Rotsztejn, H. Antioxidant Properties of Ferulic Acid and Its Possible Application. *Skin Pharmacol. Physiol.* **2018**, *31* (6), 332–336.

24. Nastić, N.; Borrás-Linares, I.; Lozano-Sánchez, J.; Švarc-Gajić, J.; Segura-Carretero, A. Comparative Assessment of Phytochemical Profiles of Comfrey (*Symphytum officinale* L.) Root Extracts Obtained by Different Extraction Techniques. *Molecules* **2020**, *25*, 837.

25. Aydin, R. 2005. Conjugated linoleic acid: chemical structure, sources and biological properties. *Turkish J. Vet. Animal Sci. 29*(2), 189-195.

26. Zolkeflee, N.K.Z.; Ramli, N.S.; Azlan, A.; Abas, F. In Vitro Anti-Diabetic Activities and UHPLC-ESI-MS/MS Profile of *Muntingia calabura* Leaves Extract. *Molecules* **2022**, *27*, 287.

27. Zhou, Y.-X.; Xia, W.; Yue, W.; Peng, C.; Rahman, K.; Zhang, H. Rhein: A Review of Pharmacological Activities. *Evid.-Based Complementary Altern. Med.* **2015**, *2015*, 1–10.

28. Qin, Z.; Zhang, B.; Yang, J.; Li, S.; Xu, J.; Yao, Z.; Zhang, X.; Gonzalez, F. J.; Yao, X. The Efflux Mechanism of Fraxetin-O-Glucuronides in UGT1A9-Transfected HeLa Cells: Identification of Multidrug Resistance-Associated Proteins 3 and 4 (MRP3/4) as the Important Contributors. *Frontiers in Pharmacology* **2019**, *10*, 496.

29. Kim, J.K.; Park, S.U. Quercetin and its Role in Biological Functions: An Updated Review. *EXCLI J.* **2018,** *17*, 856.

30. Subbiah, V.; Zhong, B.; Nawaz, M.A.; Barrow, C.J.; Dunshea, F.R.; Suleria, H.A.R. Screening of Phenolic Compounds in Australian Grown Berries by LC-ESI-QTOF-MS/MS and Determination of Their Antioxidant Potential. *Antioxidants* **2021**, *10*, 26.

31. Khan, A.K.; Rashid, R.; Fatima, N.; Mahmood, S.; Mir, S.; Khan, S.; Jabeen, N.; Murtaza, G. Pharmacological Activities of Protocatechuic Acid. *Acta Pol. Pharm.* **2015,** *72*(4), 643-650.

32. Šuković, D.; Knežević, B.; Gašić, U.; Sredojević, M.; Ćirić, I.; Todić, S.; Mutić, J.; Tešić, Ž. Phenolic Profiles of Leaves, Grapes and Wine of Grapevine Variety Vranac (*Vitis vinifera* L.) from Montenegro. *Foods* **2020**, *9*, 138.

33. Patel, D. K. Biological Importance, Therapeutic Benefit, and Medicinal Importance of Flavonoid, Cirsiliol for the Development of Remedies against Human Disorders. *Curr. Bioact. Compd.* **2022**, *18(3)*, 2-10.

34. Aguilar, T.; Loyola, C.; de Bruijn, J.; Bustamante, L.; Vergara, C.; von Baer, D.; Mardones, C.; Serra, I. Effect of Thermomaceration and Enzymatic Maceration on Phenolic Compounds of Grape Must Enriched by Grape Pomace, Vine Leaves and Canes. *Eur. Food Res. Technol.* **2015**, *242* (7), 1149–1158.

35. Du, J.; Zhong, B.; Subbiah, V.; Barrow, C.J.; Dunshea, F.R.; Suleria, H.A.R. LC-ESI-QTOF-MS/MS Profiling and Antioxidant Activity of Phenolics from Custard Apple Fruit and By-Products. *Separations* **2021**, *8*, 62.

36. Chen, Z.; Zhong, B.; Barrow, C. J.; Dunshea, F. R.; Suleria, H. A. R. Identification of Phenolic Compounds in Australian Grown Dragon Fruits by LC-ESI-QTOF-MS/MS and Determination of Their Antioxidant Potential. *Arab. J. Chem.* **2021**, *14* (6), 103151.

37. Sun, L.; Tao, S.; Zhang, S. Characterization and Quantification of Polyphenols and Triterpenoids in Thinned Young Fruits of Ten Pear Varieties by UPLC-Q TRAP-MS/MS. *Molecules* **2019**, *24*, 159.

38. Felhi, S.; Baccouch, N.; Ben Salah, H.; Smaoui, S.; Allouche, N.; Gharsallah, N.; Kadri, A. Nutritional Constituents, Phytochemical Profiles, in Vitro Antioxidant and Antimicrobial Properties, and Gas Chromatography–Mass Spectrometry Analysis of Various Solvent

Extracts from Grape Seeds (Vitis Vinifera L.). *Food Sci. Biotechnol.* **2016**, *25* (6), 1537–1544.

39. Lu, Y.; Zhu, S.; He, Y.; Peng, C.; Wang, Z.; Tang, Q. Phytochemical Profile and Antidepressant Effect of *Ormosia henryi Prain* Leaf Ethanol Extract. *Int. J. Mol. Sci.* **2019**, *20*, 3396.

40. Melo, I. S.; Santos, S. N.; Rosa, L. H.; Parma, M. M.; Silva, L. J.; Queiroz, S. C. N.; Pellizari, V. H. Isolation and Biological Activities of an Endophytic Mortierella Alpina Strain from the Antarctic Moss Schistidium Antarctici. *Extremophiles* **2013**, *18* (1), 15–23.

41. Mukai, R. Prenylation Enhances the Biological Activity of Dietary Flavonoids by Altering Their Bioavailability. *Biosci. Biotechnol. Biochem.* **2018**, *82* (2), 207–215.

42. Azam, F.; Chaudhry, B. A.; Ijaz, H.; Qadir, M. I. Caffeoyl-β-d-Glucopyranoside and 1,3-Dihydroxy-2-Tetracosanoylamino-4-(E)-Nonadecene Isolated from Ranunculus Muricatus Exhibit Antioxidant Activity. *Sci. Rep.* **2019**, *9* (1).

43. Abourashed, E. A.; El-Alfy, A. T. Chemical Diversity and Pharmacological Significance of the Secondary Metabolites of Nutmeg (Myristica Fragrans Houtt.). *Phytochem. Rev.: Proceedings of the Phytochemical Society of Europe* **2016**, *15* (6), 1035–1056.

44. Correa-Betanzo, J.; Allen-Vercoe, E.; McDonald, J.; Schroeter, K.; Corredig, M.; Paliyath, G. Stability and Biological Activity of Wild Blueberry (Vaccinium Angustifolium) Polyphenols during Simulated in Vitro Gastrointestinal Digestion. *Food Chem.* **2014**, *165*, 522–531.

45. Untergehrer, M.; Kiermaier, J.; Reintjes, S.; Heilmann, J.; Jürgenliemk, G. Identification of Phase-II Metabolites from Human Serum Samples after Oral Intake of a Willow Bark Extract. *Phytomedicine* **2019**, *57*, 396–402.

46. Ślusarczyk, S.; Cieślak, A.; Yanza, Y.R.; Szumacher-Strabel, M.; Varadyova, Z.; Stafiniak, M.; Wojnicz, D.; Matkowski, A. Phytochemical Profile and Antioxidant Activities of *Coleus amboinicus* Lour. Cultivated in Indonesia and Poland. *Molecules* **2021**, *26*, 2915.

47. Moreira, M. R.; Souza, A. B.; Soares, S.; Bianchi, T. C.; de Souza Eugênio, D.; Lemes, D. C.; Martins, C. H. G.; da Silva Moraes, T.; Tavares, D. C.; Ferreira, N. H.; Ambrósio, S.

R.; Veneziani, R. C. S. Ent-Kaurenoic Acid-Rich Extract from Mikania Glomerata: In Vitro Activity against Bacteria Responsible for Dental Caries. *Fitoterapia* **2016**, *112*, 211–216.

48. Thawabteh, A.; Juma, S.; Bader, M.; Karaman, D.; Scrano, L.; Bufo, S.A.; Karaman, R. The Biological Activity of Natural Alkaloids against Herbivores, Cancerous Cells and Pathogens. *Toxins* **2019**, *11*, 656.

49. Leyva-Jiménez, F.J.; Ruiz-Malagón, A.J.; Molina-Tijeras, J.A.; Diez-Echave, P.; Vezza, T.; Hidalgo-García, L.; Lozano-Sánchez, J.; Arráez-Román, D.; Cenis, J.L.; Lozano-Pérez, A.A.; Rodríguez-Nogales, A.; Segura-Carretero, A.; Gálvez, J. Comparative Study of the Antioxidant and Anti-Inflammatory Effects of Leaf Extracts from Four Different *Morus alba* Genotypes in High Fat Diet-Induced Obesity in Mice. *Antioxidants* **2020**, *9*, 733.

50. Scholz, E.; Heinrich, M.; Hunkler, D. Caffeoylquinic Acids and Some Biological Activities OfPluchea Symphytifolia. *Planta Med.* **1994**, *60* (04), 360–364.

51. Piazzon, A.; Vrhovsek, U.; Masuero, D.; Mattivi, F.; Mandoj, F.; Nardini, M. Antioxidant Activity of Phenolic Acids and Their Metabolites: Synthesis and Antioxidant Properties of the Sulfate Derivatives of Ferulic and Caffeic Acids and of the Acyl Glucuronide of Ferulic Acid. *J. Agric. Food Chem.* **2012**, *60* (50), 12312–12323.

52. Salehi, B.; Venditti, A.; Sharifi-Rad, M.; Kręgiel, D.; Sharifi-Rad, J.; Durazzo, A.; Lucarini, M.; Santini, A.; Souto, E.B.; Novellino, E.; Antolak, H.; Azzini, E.; Setzer, W.N.; Martins, N. The Therapeutic Potential of Apigenin. *Int. J. Mol. Sci.* **2019**, *20*, 1305.

53. De Beer, D.; Schulze, A.E.; Joubert, E.; De Villiers, A.; Malherbe, C.J.; Stander, M.A. Food Ingredient Extracts of *Cyclopia subternata* (Honeybush): Variation in Phenolic Composition and Antioxidant Capacity. *Molecules* **2012**, *17*, 14602-14624.

54. Rashid, M. I.; Fareed, M. I.; Rashid, H.; Aziz, H.; Ehsan, N.; Khalid, S.; Ghaffar, I.; Ali, R.; Gul, A.; Hakeem, K. R. Flavonoids and Their Biological Secrets. *Plant and Human Health, Volume 2* **2019**, 579–605.

55. Karthikeyan, G.; Rajendran, L.; Sendhilvel, V.; Prabakar, K.; Raguchander, T. Diversity and Functions of Secondary Metabolites Secreted by Epi-Endophytic Microbes and Their

Interaction with Phytopathogens. *Biocontrol Agents and Secondary Metabolites* **2021**, 495–517.

56. Fuloria, S.; Sekar, M.; Khattulanuar, F.S.; Gan, S.H.; Rani, N.N.I.M.; Ravi, S.; Subramaniyan, V.; Jeyabalan, S.; Begum, M.Y.; Chidambaram, K.; Sathasivam, K.V.; Safi, S.Z.; Wu, Y.S.; Nordin, R.; Maziz, M.N.H.; Kumarasamy, V.; Lum, P.T.; Fuloria, N.K. Chemistry, Biosynthesis and Pharmacology of Viniferin: Potential Resveratrol-Derived Molecules for New Drug Discovery, Development and Therapy. *Molecules* **2022**, *27*, 5072.
57. Miras-Moreno, B.; Sabater-Jara, A. B.; Pedreño, M. A.; Almagro, L. Bioactivity of Phytosterols and Their Production in Plant in Vitro Cultures. *J. Agric. Food Chem.* **2016**, *64* (38), 7049–7058.
58. Ho, M.-L.; Chen, P.-N.; Chu, S.-C.; Kuo, D.-Y.; Kuo, W.-H.; Chen, J.-Y.; Hsieh, Y.-S. Peonidin 3-Glucoside Inhibits Lung Cancer Metastasis by Downregulation of Proteinases Activities and MAPK Pathway. *Nutr. Cancer* **2010**, *62* (4), 505–516.
59. Fernandes, C. P.; Corrêa, A. L.; Lobo, J. F. R.; Caramel, O. P.; de Almeida, F. B.; Castro, E. S.; Souza, K. F. C. S.; Burth, P.; Amorim, L. M. F.; Santos, M. G.; Ferreira, J. L. P.; Falcão, D. Q.; Carvalho, J. C. T.; Rocha, L. Triterpene Esters and Biological Activities from Edible Fruits of*Manilkara Subsericea*(Mart.) Dubard, Sapotaceae. *BioMed Res. Int.* **2013**, *2013*, 1–7.
60. Zahr, A.; Alcaide, P.; Yang, J.; Jones, A.; Gregory, M.; dela Paz, N. G.; Patel-Hett, S.; Nevers, T.; Koirala, A.; Luscinskas, F. W.; Saint-Geniez, M.; Ksander, B.; D'Amore, P. A.; Argüeso, P. Endomucin Prevents Leukocyte–Endothelial Cell Adhesion and Has a Critical Role under Resting and Inflammatory Conditions. *Nature Commun.* **2016**, *7* (1).
61. Denaro, M.; Smeriglio, A.; Trombetta, D. Antioxidant and Anti-Inflammatory Activity of *Citrus* Flavanones Mix and Its Stability after In Vitro Simulated Digestion. *Antioxidants* **2021**, *10*, 140.
62. Rasul, A.; Millimouno, F. M.; Ali Eltayb, W.; Ali, M.; Li, J.; Li, X. Pinocembrin: A Novel Natural Compound with Versatile Pharmacological and Biological Activities. *BioMed Res. Int.* **2013**, *2013*, 1–9.

63. Baldisserotto, A.; Malisardi, G.; Scalambra, E.; Andreotti, E.; Romagnoli, C.; Vicentini, C.B.; Manfredini, S.; Vertuani, S. Synthesis, Antioxidant and Antimicrobial Activity of a New Phloridzin Derivative for Dermo-Cosmetic Applications. *Molecules* **2012**, *17*, 13275-13289.

64. Liang, Z.; Liang, H.; Guo, Y.; Yang, D. Cyanidin 3-*O*-galactoside: A Natural Compound with Multiple Health Benefits. *Int. J. Mol. Sci.* **2021**, *22*, 2261.

65. Guimarães, R.; Barros, L.; Dueñas, M.; Calhelha, R. C.; Carvalho, A. M.; Santos-Buelga, C.; Queiroz, M. J. R. P.; Ferreira, I. C. F. R. Nutrients, Phytochemicals and Bioactivity of Wild Roman Chamomile: A Comparison between the Herb and Its Preparations. *Food Chem.* **2013**, *136* (2), 718–725.

66. Qamar, M.; Akhtar, S.; Ismail, T.; Wahid, M.; Barnard, R.T.; Esatbeyoglu, T.; Ziora, Z.M. The Chemical Composition and Health-Promoting Effects of the *Grewia* Species—A Systematic Review and Meta-Analysis. *Nutrients* **2021**, *13*, 4565.

67. Valadares, Y. M.; Brandão, G. C.; Kroon, E. G.; Souza Filho, J. D.; Oliveira, A. B.; Braga, F. C. Antiviral Activity of Solanum Paniculatum Extract and Constituents. *Z. Naturforsch.* **2009**, *64* (11-12), 813–818.

68. Semwal, D.K.; Semwal, R.B.; Combrinck, S.; Viljoen, A. Myricetin: A Dietary Molecule with Diverse Biological Activities. *Nutrients* **2016**, *8*, 90.

69. Sari, D.R.T.; Cairns, J.R.K.; Safitri, A.; Fatchiyah, F. Virtual Prediction of the Delphinidin-3-O-Glucoside and Peonidin-3-O-Glucoside as Anti-Inflammatory of TNF-α Signaling. *Acta Inform. Med.* **2019,** *27*(3), 152.

70. Topal, F.; Nar, M.; Gocer, H.; Kalin, P.; Kocyigit, U. M.; Gülçin, İ.; Alwasel, S. H. Antioxidant Activity of Taxifolin: An Activity–Structure Relationship. *J. Enzyme Inhib. Med. Chem.* **2015**, *31* (4), 674–683.

71. Bao-Qing, W., *Salvia Miltiorrhiza*: Chemical and Pharmacological Review of a Medicinal Plant. *J. Med. Plants Res.* **2010,** *4*(25), 2813-2820.

72. Metzger, J. D. Comparison of Biological Activities of Gibberellins and Gibberellin-Precursors Native to Thlaspi Arvense L. *Plant Physiol.* **1990**, *94* (1), 151–156.

73. Yang, D.; Wang, T.; Long, M.; Li, P. Quercetin: Its Main Pharmacological Activity and Potential Application in Clinical Medicine. *Oxid. Med. Cell. Longev.* **2020**, *2020*, 1–13.

74. Murillo, A.G.; Hu, S.; Fernandez, M.L. Zeaxanthin: Metabolism, Properties, and Antioxidant Protection of Eyes, Heart, Liver, and Skin. *Antioxidants* **2019**, *8*, 390.

75. Papastamoulis, Y.; Richard, T.; Nassra, M.; Badoc, A.; Krisa, S.; Harakat, D.; Monti, J.-P.; Mérillon, J.-M.; Waffo-Teguo, P. Viniphenol A, a Complex Resveratrol Hexamer FromVitis ViniferaStalks: Structural Elucidation and Protective Effects against Amyloid-β-Induced Toxicity in PC12 Cells. *J. Nat. Prod.* **2014**, *77* (2), 213–217.

76. Walters, D.; Meurer-Grimes, B.; Rovira, I. Antifungal Activity of Three Spermidine Conjugates. *FEMS Microbiol. Lett.* **2001**, *201* (2), 255–258.

77. Karapandzova, M.; Stefkov, G.; Cvetkovikj, I.; Stanoeva, J. P.; Stefova, M.; Kulevanova, S. Flavonoids and Other Phenolic Compounds in Needles of Pinus Peuce and Other Pine Species from the Macedonian Flora. *Nat. Prod. Commun.* **2015**, *10* (6), 1934578X1501000.

78. Carlsen, S. C. K.; Pedersen, H. A.; Spliid, N. H.; Fomsgaard, I. S. Fate in Soil of Flavonoids Released from White Clover (Trifolium RepensL.). *Appl. Environ. Soil Sci.* **2012**, *2012*, 1–10.

79. Hazrati, H.; Fomsgaard, I. S.; Kudsk, P. Targeted Metabolomics Unveil Alteration in Accumulation and Root Exudation of Flavonoids as a Response to Interspecific Competition. *J. Plant Interact.* **2021**, *16* (1), 53–63.

80. Zheng, J.; Ding, C.; Wang, L.; Li, G.; Shi, J.; Li, H.; Wang, H.; Suo, Y. Anthocyanins Composition and Antioxidant Activity of Wild Lycium Ruthenicum Murr. From Qinghai-Tibet Plateau. *Food Chem.* **2011**, *126* (3), 859–865.

81. Aboulaghras, S.; Sahib, N.; Bakrim, S.; Benali, T.; Charfi, S.; Guaouguaou, F.-E.; Omari, N.E.; Gallo, M.; Montesano, D.; Zengin, G.; Taghzouti, K.; Bouyahya, A. Health Benefits and Pharmacological Aspects of Chrysoeriol. *Pharmaceuticals* **2022**, *15*, 973.

82. Zhu, L.; Zhang, Y.; Deng, J.; Li, H.; Lu, J. Phenolic Concentrations and Antioxidant Properties of Wines Made from North American Grapes Grown in China. *Molecules* **2012**, *17*, 3304-3323.
